# Plasma alpha B crystallin as potential biomarker for predicting pre-operative seizures in glioma

**DOI:** 10.1186/s12883-024-03740-x

**Published:** 2024-07-06

**Authors:** Yongsheng Xie, Zengxin Qi, Yusheng Tong, Nan Zhou

**Affiliations:** 1 Department of Neurosurgery, Huashan Hospital, Shanghai Medical College, National Centre for Neurological Disorders, Shanghai Key Laboratory of Brain Function and Restoration and Neural Regeneration, Shanghai Clinical Medical Centre of Neurosurgery, Neurosurgical Institute of Fudan University, Shanghai, 200040 China; 2 State Key Laboratory of Medical Neurobiology and MOE Frontiers Centre for Brain Science, School of Basic Medical Sciences, Institutes of Brain Science, Fudan University, Shanghai, 200040 China

**Keywords:** Crystallin, Plasma biomarker, Epilepsy, Glioma

## Abstract

**Purpose:**

Glioma-associated epilepsy affects a significant proportion of glioma patients, contributing to disease progression and diminished survival rates. However, the lack of a reliable preoperative seizure predictor hampers effective surgical planning. This study investigates the potential of Alpha B crystallin protein (CRYAB) plasma levels as a predictive biomarker for epilepsy seizures in glioma patients.

**Methods:**

Plasma samples were obtained from 75 participants, including 21 glioma patients with pre-operative epilepsy, 14 glioma patients without pre-operative epilepsy, and 21 age- and sex-matched control subjects. Additionally, 11 idiopathic epilepsy patients and 8 intractable epilepsy patients served as positive disease control groups. The study utilized ELISA to accurately quantify the circulating levels of CRYAB in the plasma samples of all participants.

**Results:**

The analysis revealed a significant reduction in plasma CRYAB levels in glioma patients with pre-operative epilepsy and idiopathic epilepsy. The receiver operating characteristic (ROC) curve analysis displayed an impressive performance, indicating an AUC of 0.863 (95% CI, 0.810–0.916) across the entire patient cohort. Furthermore, plasma CRYAB levels exhibited a robust diagnostic capability, with an AUC of 0.9135, a sensitivity of 100.0%, and a specificity of 73.68%, effectively distinguishing glioma patients with preoperative epilepsy from those without epilepsy. The Decision Curve Analysis (DCA) underscored the clinical relevance of plasma CRYAB levels in predicting pre-operative epilepsy in glioma.

**Conclusion:**

The findings imply that the reduced levels of CRYAB may assist in prediction of seizure occurrence in glioma patients, although future large-scale prospective studies are warranted.

**Supplementary Information:**

The online version contains supplementary material available at 10.1186/s12883-024-03740-x.

## Introduction

The incidence of glioma, the most prevalent malignant tumors in the central nervous system, exceeds 60 million worldwide. Epilepsy associated with glioma frequently arises in 40–70% of glioma patients. Epilepsy markedly reduces the quality of life for these patients and leads to considerable economic and neurocognitive decline. Additionally, epilepsy has been associated with amplified disease progression and decreased survival rates in patients with glioma [1, 2]. In cases of glioma, the recurrence or worsening of seizures after initial antitumor therapy signifies progression in roughly two-thirds of patients [3, 4]. Attaining seizure freedom is crucial for surgical planning; however, a dependable predictor of preoperative seizures has not yet been identified, highlighting the urgent requirement for a biomarker capable of predicting epilepsy before initiating treatment.

The Alpha B crystallin protein (CRYAB) is a extensively studied and prominent member of the small chaperone protein family within the heat shock protein group [5]. Elevated levels of CRYAB mRNA or protein have been associated with various pathological conditions, particularly in the brain. These conditions include Alexander’s disease, a rare hereditary degenerative brain disorder in children [6]; diffuse Lewy body disease, one of the major neurodegenerative dementing diseases in humans [7, 8]; tuberous sclerosis, an autosomal dominant disorder characterized by benign tumor growth and malformations in the skin, central nervous system, kidney, and other organs [9, 10]; ballooned neurons observed in various disorders such as Creutzfeldt-Jacob disease [11, 12]; and glial tumors [13–15]. Regarding epilepsy, elevated levels of immunoreactive CRYAB in astrocytes and oligodendrocytes have been identified in pediatric epileptic patients [16–18]. Animal studies have shown that repeated seizure induction upregulates CRYAB in kindling models in rats [19, 20], mice [21], and canines [20]. Considering the association between repeated kindled seizures and increased seizure susceptibility along with reduced seizure thresholds, the upregulation of CRYAB may indicate its involvement in the pathophysiological mechanisms underlying epilepsy development. Given previous observations of activated crystallin in epileptogenic tissue [16–18] as well as its high expression in glioma tissue [13–15], it is reasonable to propose that CRYAB could potentially serve as a metabolic marker for epileptic activity when measured in the plasma of patients with glioma. This study aimed to investigate plasma levels of CRYAB as a biomarker for predicting epilepsy seizures among patients with glioma.

## Method

### Patients

Patients were classified into five groups: the pre-seizure group, including patients with glioma who experienced frequent seizures during the pre-operation period; the no-seizure group, comprising patients with glioma who did not experience any seizure events or only a single event during the pre-operation period; the intractable epilepsy group, consisting of non-brain tumor patients with a long course of epilepsy; the idiopathic epilepsy group, serving as a positive epileptic disease control; and finally, the control group, consisting of age- and sex-matched healthy subjects serving as negative controls. The study obtained approval from the Ethics Committee of Huashan Hospital, Fudan University. Histological classification was conducted in accordance with the 2016 WHO standards for grading central nervous system tumors [22], encompassing grades I to IV. The predominant histological types included astrocytoma, oligodendroglioma, and glioblastoma. The diagnosis of epileptic seizures was established based on the 2017 International League Against Epilepsy (ILAE) criteria [23], with semiological seizure classification performed through preoperative evaluation. Additionally, a majority (84%) underwent intraoperative electrocorticography (iECoG) during awake craniotomy-assisted surgery. The exclusion criteria were as follows: (1) a history of previous treatment for glioma or other intracranial diseases; (2) a history of previous or concomitant malignant disease; and (3) inadequate liver or renal function. Demographic and clinical data of all enrolled patients were retrospectively obtained through a comprehensive review of their medical records. The collected clinical data included patient demographics, epileptic seizure history, tumor location, tumor grade (according to the revised WHO 2016 classification) [16], p53 expression levels, IDH1 mutation status, Ki-67 proliferation index values, CD34 expression levels, olig-2 expression levels, and ATRX expression levels (refer to Tables 1 and 2 for detailed information).

**Table 1:** Demographic and clinical characteristics of the glioma patients

		Total	Pre-epilepsy	Non-epilepsy	*P*
		*N* = 35	*N* = 21	*N* = 14	
Gender (male/female)		22/13	16/5	6/8	0.075
Age at Diagnosis		49 (29–73)	47(29–70)	51(29–73)	0.465
WHO Grade					0.258
II		13(37%)	10 (48%)	3 (21%)	
III		3(9%)	2 (10%)	1 (7%)	
IV		19(54%)	9 (43%)	10(71%)	
Tumor Laterality					0.036
Left		10(29%)	8(38%)	2(14%)	
Right		13(37%)	4(19%)	9(64%)	
Midline		0	0	0	
Tumor Location					0.602
Frontal		15(43%)	9(43%)	6(43%)	
Parietal		0	0	0	
Temporal		5(14%)	3(14%)	2(14%)	
Occipital		1(3%)	0	1(7%)	
Other (Deep nuclei or thalamic)		1(3%)	0	1(7%)	
Tumor pathology (2016 WHO)					0.381
II	Diffuse astrocytoma, IDH1 ^mut^	3(9%)	2 (10%)	1 (7%)	
	Oligodendro, IDH1 ^mut^ and 1p/19q-codeleted	4(11%)	3 (14%)	1(7%)	
	Oligodendro IDH1 ^wt^	0	0	0	
III	Diffuse astrocytoma, IDH1 ^wt^	0	0	0	
	Anaplastic astrocytoma, IDH1 ^wt^	0	0	0	
	Anaplastic astrocytoma, IDH1 ^mut^	0	0	0	
	Oligodendro anaplastic, IDH1 ^mut^ and 1p/19q- codeleted	2 (6%)	2 (10%)	0	
IV	Glioblastoma, IDH1 ^wt^	14 (40%)	6 (29%)	8 (57%)	
	Glioblastoma, IDH1 ^mut^	1(3%)	1(5%)	0	
	Diffuse astrocytoma ^mut^	2 (6%)	1 (5%)	1(7%)	

**Table 2:** Immunohistochemical indices of the study population

	Total	Pre-epilepsy	Non-epilepsy	*P* value
	*N* = 35	*N* = 21	*N* = 14	
GFAP, N (%)				
(i)(-)	0 (0)	0 (0)	0 (0)	
(ii)(+)	35 (100%)	21 (100%)	14 (100%)	
IDH-1, N (%)				0.122
(i)(-)	18 (51%)	8 (38%)	10 (71%)	
(ii)(+)	14 (40%)	10 (48%)	4 (29%)	
Missing	3 (9%)	3 (14%)	0 (0)	
Olig-2, N (%)				
(i)(-)	0 (0)	0 (0)	0 (0)	
(ii)(+)	35 (100%)	21 (95%)	14 (100%)	
P53,N (%)				0.185
(i)(-)	10 (28.5%)	8 (38%)	2 (14%)	
(ii)(+)	24 (68.5%)	12 (57%)	12 (86%)	
Missing	1(3%)	1(5%)	0(0)	
ATRX, N (%)				0.776
(i)(-)	4 (11%)	3 (14%)	1 (7%)	
(ii)(+)	30 (86%)	17 (81%)	13 (93%)	
Missing	1(3%)	1(5%)	0(0)	
H3K27ME3, N (%)				0.714
(i)(-)	1 (4.5%)	1 (8%)	0 (0)	
(ii)(+)	20 (91%)	11 (92%)	9 (90%)	
Missing	1 (4.5%)	0 (0)	1 (10%)	
Ki-67, N (%)				<0.001
(i)Low	22 (63%)	18 (85.7%)	4 (29%)	
(ii)Medium	9 (26%)	1 (4.8%)	8 (57%)	
(iii)High	4 (11%)	2 (9.5%)	2 (14%)	
FuBP1, N (%)				1.000
(i)(-)	1 (3%)	1 (5%)	0 (0)	
(ii)(+)	29 (83%)	17 (81%)	12 (86%)	
Missing	5 (14%)	3 (14%)	2 (14%)	
BRAFV600E, N (%)				0.019
(i)(-)	26 (74%)	19 (90%)	7 (50%)	
(ii)(+)	6 (17%)	1 (5%)	5 (36%)	
Missing	3 (9%)	1 (5%)	2 (14%)	
Nestin, N (%)				0.122
(i)(-)	8 (23%)	7 (33.3%)	1 (7%)	
(ii)(+)	23 (66%)	11 (52.4%)	12 (86%)	
Missing	4 (11%)	3 (14.3%)	1 (7%)	

### Plasma collection

Plasma samples are obtained during the patient’s first routine blood test after admission. A 2 ml venous blood sample was collected from each patient. The samples were drawn into tubes containing EDTA and immediately centrifuged at room temperature (centrifugation at 2,500 × g) to separate the plasma. The resulting supernatant was then transferred into RNase-free EP tubes and stored at -80˚C until use.

### ELISA measurements of plasma CRYAB

The sample and CRYISA Kit (Human) (OKEH04152, CA, Aviva Systems Biology) were equilibrated to room temperature prior to use and promptly utilized. 1 mL of Sample Diluent was added to the lyophilized CRYAB standard containing 10 ng for the preparation of standards. The standard samples were diluted at seven concentrations according to the instructions. The 1X Biotinylated CRYAB Detector Antibody was prepared immediately before use by diluting 100X Biotinylated CRYAB Detector Antibody with Detector Antibody Diluent in a ratio of 1:100. The wash buffer solution was prepared by transferring the entire contents (30 mL) from the Wash Buffer bottle into a clean vessel containing ultra-pure water (720 ml), with a total volume of > 1,000 mL. Subsequently, two replicates each were added for samples and standards by dispensing 100 µL into microplate wells. The plate was covered with a well plate sealer and incubated at 37℃ for a duration of 2 h. Subsequently, the plate sealer was removed and the liquid present in the wells was discarded. To ensure thorough drying, any residual liquid within the wells was carefully blotted by tapping them upside down onto paper toweling. 100 µL of prepared 1X Biotinylated CRYAB Detector Antibody was added to each well. The wells were covered with a well-plate sealer and incubated at 37℃ for 60 min. Any remaining liquid was blotted. The plate were washed with 1X Wash Buffer, followed by the addition and removal of 300 µL wash buffer after each one-minute incubation period. 100 µL of prepared 1X Avidin-HRP Conjugate was added to each well, followed by covering the plate with a sealer and incubating it at 37℃ for 60 min. The excess liquid was absorbed by blotting. The plate was subjected to five rounds of washing with 1X Wash Buffer, with the addition and removal of excess wash buffer after each one-minute incubation period. The addition of 90 µL of TMB Substrate was performed in each well. The microplate was then sealed using a plate sealer and incubated at 37℃ without exposure to light for a duration of 30 min. Following this, the addition of 50 µL of Stop Solution was carried out in each well. The optical density (O.D.) absorbance was immediately measured at 450 nm using a microplate reader. Subsequently, the mean values of the obtained results were calculated, and the coefficient of variation (CV) was determined for each parameter. This analysis revealed an intra-assay CV of 4.31% and an inter-assay CV of 8.4% for CRAYB.

### Statistical analysis

Continuous variables were reported as mean and standard deviation, while categorical variables were presented as counts (percentages). Fisher’s exact test was employed to compare the presence and absence of preoperative seizure groups for tumor grade, tumor side, tumor pathology, IDH1 mutation, ARTX retention, MGMT gene promoter methylation, p53 expression, and Ki67. A multivariate analysis was conducted including all factors analyzed in the univariate analysis. SPSS 17.0 software (IBM Corporation, Armonk, NY) was utilized for all statistical analyses. All tests were two-sided and a significance level of *p* < 0.05 was considered statistically significant. To evaluate the discriminative ability of plasma CRYAB changes in distinguishing between subjects with pre-seizures and those without seizures in glioma patients, a receiver operating characteristic (ROC) curve and the area under the ROC curve (AUC) were calculated.

## Results

### Baseline clinical characteristics of participants and experimental data

In the present study, plasma samples were obtained from 75 participants, including 21 glioma patients with pre-operative epilepsy (G + E), 14 glioma patients without pre-operative epilepsy (G-E), and 21 age- and sex-matched control subjects (control) recruited for this study. Additionally, 11 idiopathic epilepsy (E) patients and 8 intractable epilepsy patients (IE) were included as positive disease control groups. The circulating levels of CRYAB were successfully quantified in the plasma samples of all patients by ELISA. No significant differences in gender and age were found among the five groups, except for the age of the intractable epilepsy groups, which was younger than that of the other groups (see Supplement Table 1). The demographic and baseline characteristics were obtained from clinical or pathologic records. The detailed demographic and clinical characteristics of glioma subjects were listed in Table 1.

### Plasma CRYAB was significantly decreased in glioma patients with pre-operative epilepsy and epilepsy

**Fig. 1:**
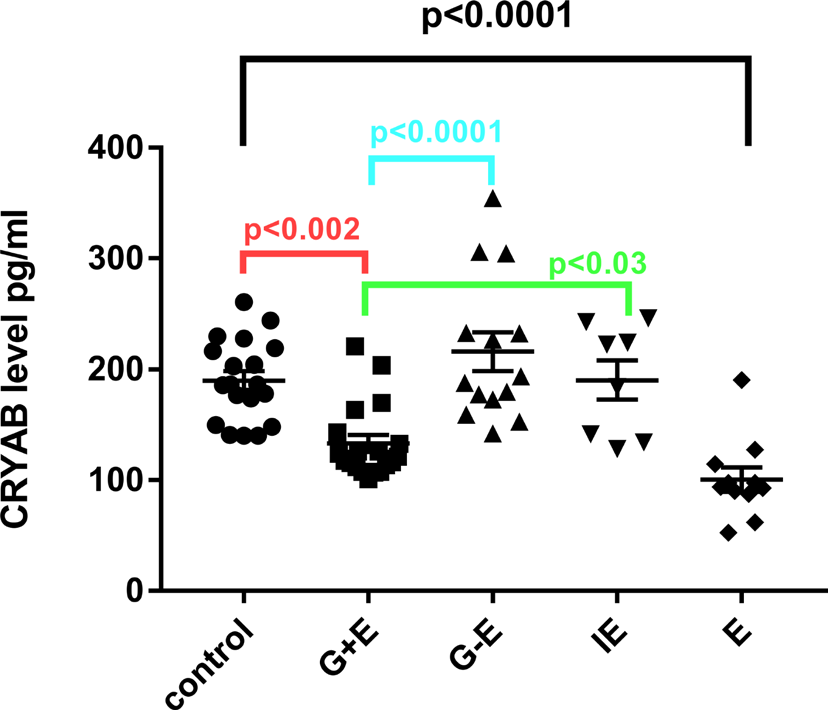
Plasma alpha B crystallin (CRYAB) in glioma with preoperative epilepsy. ELISA measurement of CRYAB in glioma patients with preoperative epilepsy (G + E), glioma without preoperative epilepsy (G-E), the patients with intractable epilepsy (IE) and epilepsy (E) patients. The plasma CRYAB levels in epilepsy groups were significant decreased compared with other four groups (*p* < 0.0001)

We initially investigated differences in the levels of plasma CRYAB among five groups; ELISA measurements were conducted on the subjects mentioned above. ANOVA analysis revealed significant differences in plasma CRYAB levels among the five groups (F = 15.12, *p* < 0.0001). Tukey’s multiple comparisons test indicated that the lowest levels of plasma CRYAB were found in the idiopathic epilepsy patients (100.5 ± 10.95 pg/ml). Interestingly, plasma CRYAB levels were significantly lower in the G + E group (133.1 ± 7.45 pg/ml) than in the G-E group (215.9 ± 17.28 pg/ml) (*p* < 0.0001) (see Fig. 1). However, there was no difference in the levels of plasma CRYAB between the G + E and IE groups (190.3 ± 17.61 pg/ml) (*p* > 0.05) (see Fig. 1).

### Clinical significance of plasma CRYAB as a potential diagnostic biomarker

**Fig. 2:**
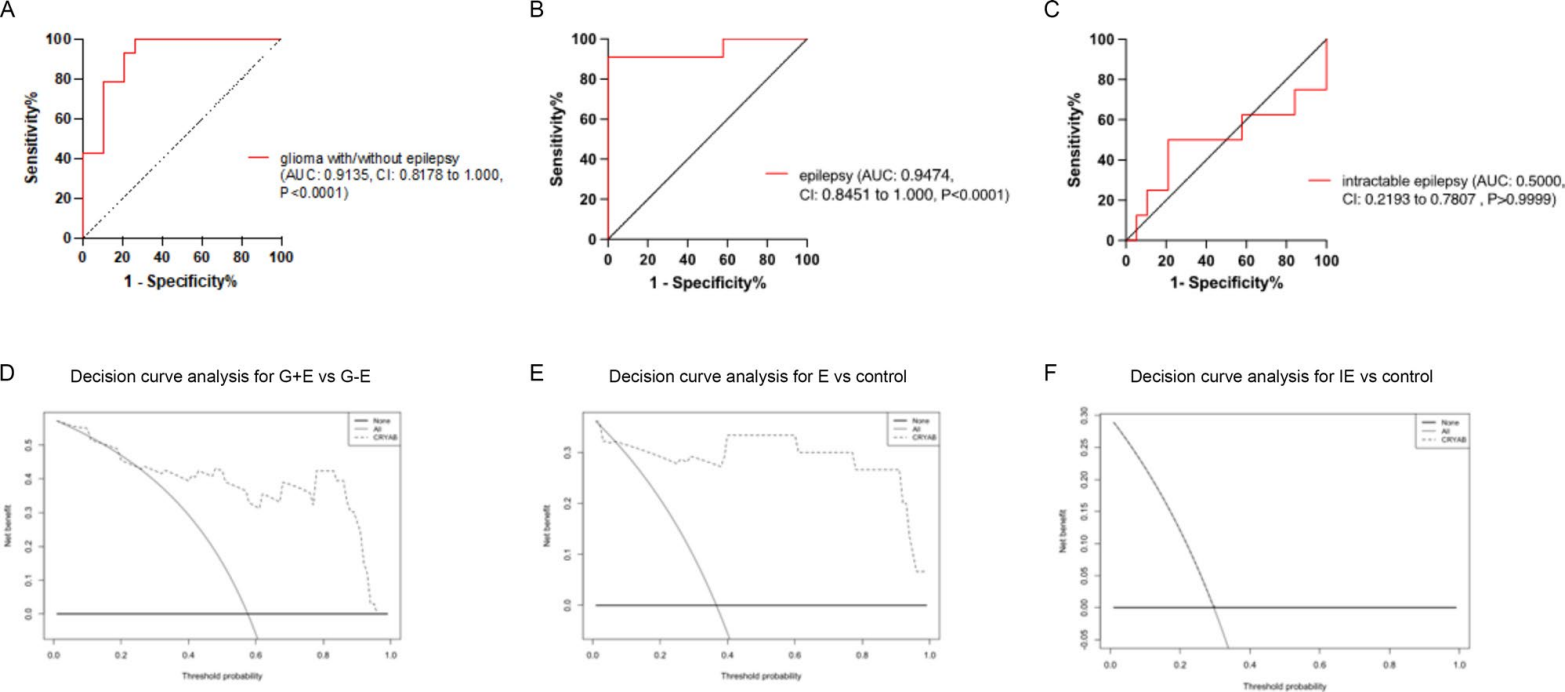
ROC curve analysis and decision curve analysis in glioma with preoperative epilepsy. **A**: A receiver operating characteristic (ROC) curve analysis shows that the concentration of CRYAB had a strong diagnostic power in discriminating glioma with epilepsy from glioma without epilepsy. **B**: and had a strong diagnostic power in discriminating epilepsy from control. **C**: ROC curve analysis shows that the concentration of CRYAB had no diagnostic power in discriminating intractable epilepsy from control. **D**: Decision curve analysis highlights the clinical benefit for the prediction of preoperative epilepsy by the decreased circulating levels of plasma CRYAB in the glioma patient with (G + E) or without preoperative epilepsy (G-E). **E**: Decision curve analysis highlights the clinical benefit for the prediction of epilepsy by the decreased circulating levels of plasma CRYAB in the epilepsy patient. **F**: The decision curves revealed that the prediction model add no net benefit for prediction intractable epilepsy (IE) by the circulating levels of plasma CRYAB

To assess the clinical relevance of plasma CRYAB levels in distinguishing between glioma patients with pre-operative epilepsy and those without, ROC curve analysis was performed. Illustrated in Fig. 2, the analysis demonstrated excellent predictive performance for epilepsy type, with an AUC of 0.863 (95% CI, 0.810–0.916) in the entire patient cohort. ROC analysis indicated that plasma CRYAB levels exhibited robust diagnostic capability (AUC of 0.9135, best cutoff value of CRYAB with sensitivity and specificity of 100.0% and 73.68% respectively) in discriminating between glioma with epilepsy and glioma without epilepsy (Fig. 2A). Correspondingly, there was strong diagnostic potential (AUC of 0.9474, best cutoff value of CRYAB with sensitivity and specificity of 90.91% and 100% respectively) in distinguishing idiopathic epilepsy from the control group (Fig. 2B). Conversely, the ROC curve analysis revealed that plasma CRYAB levels had no diagnostic power (AUC of 0.5000) in distinguishing intractable epilepsy from control subjects (Fig. 2C).

### Determining the predictive accuracy of the CRYAB-signature in the glioma with pre-operative epilepsy using ROC analysis

Decision curve analysis (DCA) emphasizes the clinical benefits of predicting pre-operative epilepsy and managing the disease through plasma levels of CRYAB. Decision curves of the multivariate prediction models for glioma patients with pre-operative epilepsy were based on (A) plasma levels of CRYAB and (B) the multivariable model integrating circulating levels of CRYAB. Net benefit is plotted across various threshold probabilities. Given the clinical significance of plasma CRYAB levels in predicting pre-operative seizures, we sought to evaluate their clinical utility in patient treatment. Decision curve analysis, conducted following Vickers et al., underscored the clinical benefit of tested CRYAB levels in predicting treatment for glioma patients with pre-operative epilepsy (Fig. 2D) [24]. In this context, the univariate analysis demonstrated the superior clinical value of circulating CRYAB levels in glioma patients with pre-operative epilepsy. The DCA of the G + E and G-E groups indicated that a threshold probability of plasma CRYAB levels above 30% was clinically significant (Fig. 2D).

### Factors affecting plasma CRYAB and clinic pathological characteristics

The above findings suggest that plasma CRYAB may be a useful diagnostic biomarker for predicting epilepsy during the preoperative period in glioma patients. To identify factors potentially associated with tumor-related epilepsy, demographic and tumor information was compared between patients with and without seizures (see Table 1 for a complete list). The chi-square test revealed no statistically significant differences in age at diagnosis, WHO grade, tumor location, and tumor pathology (2016 WHO) between glioma patients with and without pre-operative seizures (Table 1). Analysis of immunohistochemical indices and IDH1 mutation results showed no differences in GFAP, Olig-2, P53, ATRX, H3K227ME3, FuBP1, BRAFV600E, and Nestin between glioma patients with and without pre-operative seizures (Table 2). Although more patients exhibited lower expression of Ki-67 (low, ≤ 5%; medium, 6–20%; high, > 20%) in the group with pre-operative seizures compared to those without, no difference was observed in plasma CRYAB levels between subjects with low Ki-67 expression (136.7 ± 10.29) and those with high Ki-67 expression (133.8 ± 5.73). Subsequent univariate and multivariate analyses indicated no differences in these clinicopathological features between the two matched groups. It is worth mentioning that, due to the limited sample size, the study lacked sufficient statistical power to evaluate differences in tumor molecular features between the G + E and G-E groups.

**Fig. 3:**
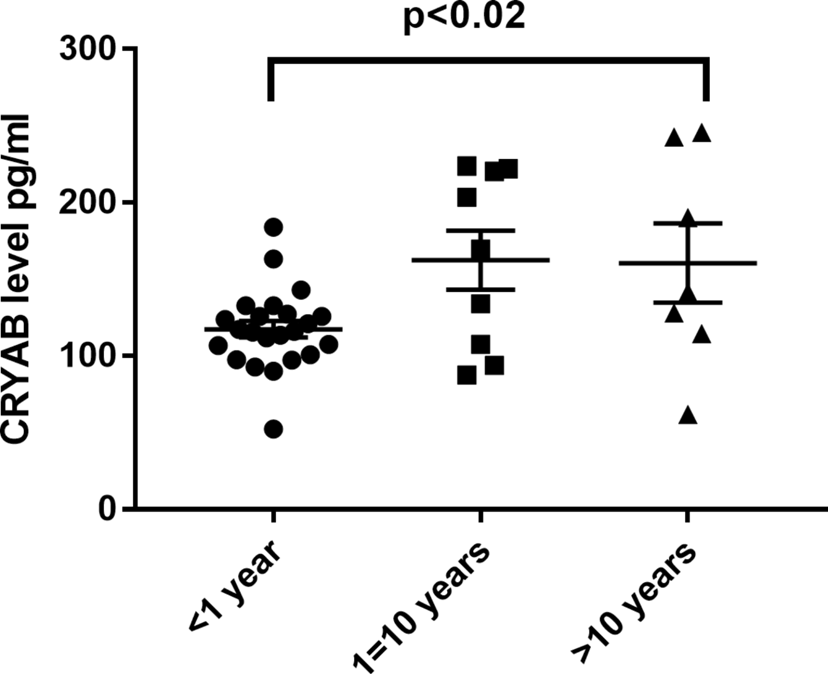
Alterations in plasma alpha B crystallin (CRYAB) during the course of epilepsy. The levels of CRYAB were significant decreased in the patients with epileptic course shorter than one year when all of the epileptic patients including glioma with preoperative seizure, intractable epilepsy and epilepsy were pooled together

**Fig. 4:**
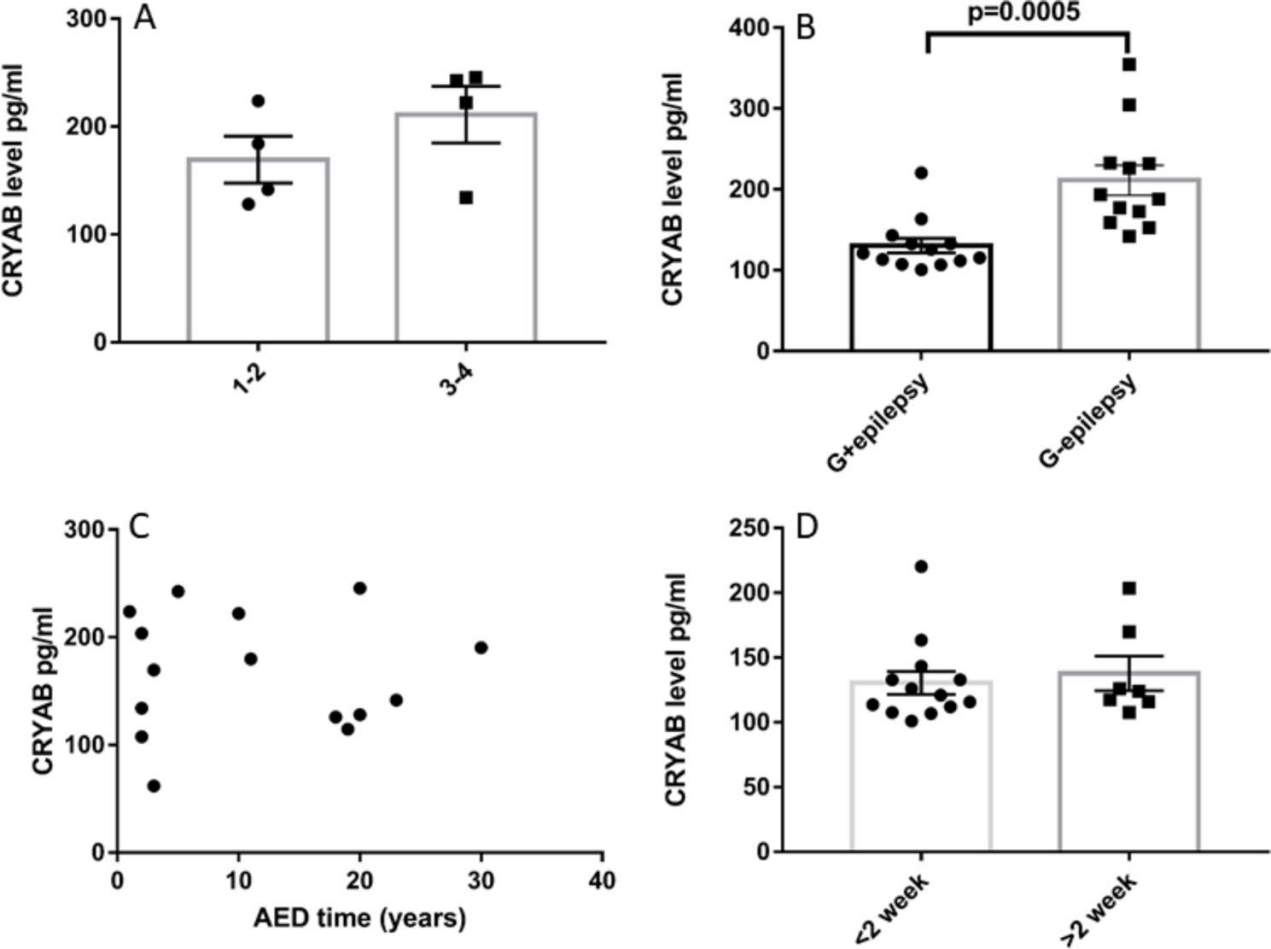
The administration of AEDs does not have any impact on the plasma levels of CRYAB. **A**: No influence of the number of AEDs on CRYAB levels. 1–2: using 1 or 2 AEDs, 3–4: using 3 or 4 AEDs, *N* = 4 in each group, *P* = 0.26; **B**: Two weeks used of AEDs had no effect on CRYAB levels in glioma patients. The administration of AEDs occurred within a two-week timeframe for both patient groups; however, glioma patients with epilepsy (G + E *n* = 13) exhibited lower levels of CRYAB compared with glioma patients without epilepsy (G-E *n* = 12) (*p* = 0.0005); **C**: No relationship between the duration of AEDs used and CRYAB levels. There was no correlation between the duration of AEDs ranged from 1 year to 30 years and the levels of CRYAB. *r* = 0.02, *p* = 0.95. The patients consisted of glioma with epilepsy (*n* = 5) intractable epilepsy (7) and idiopathic epilepsy (*n* = 3); **D**: The duration of AED administration, whether shorter or (*n* = 13) longer than two weeks (*n* = 7), did not demonstrate any significant differences in glioma patients with epilepsy. *P* = 0.64

We subsequently assessed the correlation between CRYAB and the epileptic state. The comparison among the five groups revealed that plasma CRYAB levels were lowest in idiopathic epilepsy patients, followed by glioma with pre-operative epilepsy. Interestingly, the plasma CRYAB levels in IE patients mirrored those of the control cohort (see Fig. 1). Our findings indicated no significant correlation between plasma CRYAB levels and clinical features, such as seizure onset and the presence or absence of grand mal (refer to Table 3). Intriguingly, we observed a significant association between plasma CRYAB levels and the duration of epilepsy (refer to Table 3). Patients with an epileptic course of less than one year exhibited the lowest levels of plasma CRYAB (refer to Fig. 3). Additionally, the number of anti-seizure medication ASM used was also linked to plasma CRYAB levels (refer to Table 3). The influence of the number of ASMs on CRYAB levels was investigated by comparing patients using 1 or 2 (*n* = 4) with those using 3 or 4 ASMs within the intractable epilepsy group (*n* = 4). As showed in Fig. 4A, there was no difference in CRYAB levels. *p* = 0.26 indicating the number of administration of ASMs might not effect on the CRYAB levels. The ASMs is routinely employed in glioma patients period to surgery in our hospital as prophylactic administration, regardless of seizure occurrence. Specifically, we compared the CRYAB levels between glioma patients with or without epilepsy who all taken ASMs within a two-week timeframe. Again, the CRYAB levels was significant lower in glioma patients with epilepsy than that of without epilepsy indicating that CRYAB levels is more disease dependent than the administration of ASMs (Fig. 4B). Furthermore, the correlation between the duration of ASMs used and CRYAB levels were performed by Spearman analysis. The three groups of patients who taken ASMs more than one years were included (*n* = 15). There was no correlation between the duration of ASMs and the levels of CRYAB. *r* = 0.02, *p* = 0.95 (Fig. 4C). In addition, the duration of ASMs administration, whether shorter or (*n* = 13) longer than two weeks (*n* = 7), did not demonstrate any significant differences in the group of glioma patients with epilepsy. *P* = 0.64 (Fig. 4D). To further assess whether the lower quartile of plasma CRYAB levels in the G-E group is attributed to their treatment with ASMs and if there is an overlap between the lower quartile levels and those of the G + E groups receiving ASMs, an analysis was conducted on the first quartile and median levels of CRYAB across all groups (Supplementary Table 3). In the lower quartile of the G-E group, three patients underwent prophylactic administration of 1 or 2 ASM prior to surgery. Among them, patient G22 received levetiracetam for a duration of two days, patient G26 received both levetiracetam and valproate for two days, and patient G27 received both levetiracetam and valproate for three days. Their plasma CRYAB levels were recorded as 152.88, 159.33, and 142.05 respectively; these values did not overlap with the median level observed in the G + E group. These results indicate that CRYAB levels is more disease dependent than the administration of ASMs. Regarding tumor treatment, a total of four patients with G + E and two patients with G-E received radiotherapy and chemotherapy. The plasma CRAYB level was monitored within the respective groups.

**Table 3:** Demographics and seizure related clinical characteristics of study cohort

	G + E	IE	Epilepsy	*P*
Male: Female	16:5	6:2	10:1	0.661
Age (range)	47 ± 3	47 ± 2	28 ± 6	0.006
Case number	21	8	11	
Course of epilepsy				0.001
< 1 year 1–10 year > 10 year	17 4 0	1 3 4	6 2 3	
Onset of seizure (years)	46.3	14.9	39.5	0.001
Bilateral tonic-clonic Yes: No	8:13	5:3	5:6	0.498
Number of ASMs 1: 2	8:13	1:7	8:3	0.037

*Note* G + E: Glioma-related preoperative seizures, IE intractable epilepsy; Laterality: Laterality of epileptogenic zone; ASMs: Number of anti-seizure medication at inclusion, and 2 including also more than two kinds of ASMs

## Discussion

This study primarily aimed to compare associations of plasma CRYAB levels with preoperative seizures in glioma patients. Our findings reveal that decreased plasma levels of CRYAB are observed in glioma patients with pre-operative seizures in comparison to those without pre-operative seizures. The association between decreased CRYAB levels and epilepsy was further supported by the identification of the lowest levels of plasma CRYAB in idiopathic epilepsy patients. Moreover, the decision curve analysis underscored the clinical utility of tested CRYAB levels in predicting treatment for glioma patients with pre-operative epilepsy.

The decreased plasma CRYAB in epilepsy is of particular interest, as early studies describe an increase in the expression of CRYAB in astrocytes and oligodendrocytes of the neocortex, hippocampus, and amygdala in pediatric epilepsy patients [16–18]. Notably, low plasma levels of heat shock 70 protein, a family member of CRYAB, were also found after an acute stroke to be predictive of epilepsy in a large-scale study [25]. These discrepancies may be attributed to the different protein distribution in plasma and the central nervous system. A well-known example is that amyloid A beta 40–42 is typically decreased in the plasma, while it is increased in cerebrospinal fluid in Alzheimer’s disease [26]. In addition, it was surprising that the plasma level of CRYAB in intractable epilepsy patients was high compared to the glioma with epilepsy and idiopathic epilepsy groups. The reason for this may be that among the eight cases of IE, only one case had a duration of epilepsy lasting for less than one year while the remaining IE patients had a duration exceeding ten years (Table 3). As showed in Fig. 3, the patients who had longer duration of epilepsy showed the higher levels of CRYAB (Fig. 3). The hypothesis that plasma CRYAB levels may serve as an indicator of epilepsy duration is highly intriguing, necessitating a large-scale prospective study. Nevertheless, additional large-scale studies are necessary in the future to verify these results.

CRYAB is a critical element of the tumor microenvironment, positively correlated with immune checkpoints, tumor mutational burden, and influencing tumor prognosis in human cancers [27, 28]. In glioblastoma conditions, brain CRYAB levels are heightened [14, 29]. Studies have consistently reported elevated levels of CRYAB in GBM specimens [4, 10, 13, 14, 30, 31], establishing the role of CRYAB in glioma genesis [32]. Interestingly, the increased expression of CRYAB is considered to prolong the survival of head and neck squamous cell carcinoma under hypoxic conditions [33].

To mitigate potential confounding factors that might impact the experimental results, we investigated the influence of clinic pathology and molecular factors on plasma CRYAB levels. No disparities were found in the clinical characteristics of the glioma patients, including WHO grade, tumor location, and the identified molecular classification (WHO 2016), between the glioma patients with and without pre-operative seizures (Table 1). The immunohistochemical indices of the study population showed no significant differences, apart from the expression of the Ki67 proliferation index (Table 2). A higher Ki67 labeling index may predict a greater likelihood of pre-operative seizures in glioma patients [34–36]. However, we did not observe a difference in plasma CRYAB levels between the low and high expression of the Ki67 groups (see Supplement Fig S1). This lack of differentiation might be associated with the variance in Ki67 expression between low and high-grade glioma, which were not subdivided into subgroups due to limitations in the number of cases in the analysis. A more extensive sample size and extended follow-up period are necessary to establish a more reliable conclusion.

Our study has several limitations. First, a relatively small number of patients were enrolled. Second, CRYAB levels were measured in several different forms of glioma, such as diffuse astrocytoma, oloigodendroglioma, anaplastic astrocytoma, and glioblastoma. Furthermore, we only measured the plasma levels of CRYAB, while other potential sources of CRYAB, such as exosomes and tissue, should also be measured. Additionally, factors like age and onset of seizures may be confounding factors affecting the plasma levels of CRYAB. The mean age at the first seizure was younger in the epilepsy group compared with the G + E and IE groups (Table 3). Regarding medication, prophylactic administration of ASM is routinely employed in our hospital for glioma patients prior to surgery. As depicted in Fig. 4, the timing of ASM administration within a two-week period does not impact the levels of CRYAB in glioma patients. Furthermore, there is no correlation between long-term usage of ASMs and CRYAB levels in glioma patients with epilepsy, intractable epilepsy, and idiopathic epilepsy (Fig. 4).

## Conclusion

In summary, we discovered an association between plasma CRYAB levels and the presentation of preoperative seizures in glioma. The predictive value of CRYAB can be further confirmed by utilizing a larger sample size, implementing a validation testing set, or conducting cross-validation.

### Electronic supplementary material

Below is the link to the electronic supplementary material.

Supplementary Material 1

Supplementary Material 2

Supplementary Material 3

Supplementary Material 4

## Data Availability

The datasets used and/or analyzed during the current study are available from the corresponding author on reasonable request.
