# Supplementary material for: Plasma alpha B crystallin as potential biomarker for predicting pre-operative seizures in glioma
Source: BMC Neurol. 2024 Jul 6;24:237. doi: 10.1186/s12883-024-03740-x (PMC11227141; doi:10.1186/s12883-024-03740-x)
Supplement: Supplementary file 1 — Supplementary Material 1 [file 12883_2024_3740_MOESM1_ESM.docx]

**Supplementary Table 1.**

**Demographic and clinical data for the cohort**

|  | **Pre-epilepsy with glioma** | **Non-epilepsy with glioma** | **Primary epilepsy** | **Intractable**  **epilepsy** | **Normal controls** |
| --- | --- | --- | --- | --- | --- |
|  | **N=21** | **N=14** | **N=11** | **N=8** | **N=21** |
| Gender (male/female) | 16/5 | 6/8 | 16/3 | 6/2 | 14/7 |
| Age at Diagnosis (range) | 47(29-70) | 54(29–73) | 45(25-78) | 28 (9-39) | 51(32-76) |
