# Supplementary material for: Plasma alpha B crystallin as potential biomarker for predicting pre-operative seizures in glioma
Source: BMC Neurol. 2024 Jul 6;24:237. doi: 10.1186/s12883-024-03740-x (PMC11227141; doi:10.1186/s12883-024-03740-x)
Supplement: Supplementary file 2 — Supplementary Material 2 [file 12883_2024_3740_MOESM2_ESM.docx]

Table S2. Predictors of basic clinical, pathological and genetic characters

Variables Pre-epilepsy Non-epilepsy P value

Total 21 14

Age (mean) 47 51

WHO grade (paired data) 0.465

Ⅱ 8 (38 %) 2 (14.3 %)

Ⅲ 2 (9.5%) 1 (7.1 %)

Ⅳ 9 (43 %) 9 (64.3 %)

Missing 2(9.5%) 2(14.3%)

**IDHmut** (paired data) 0.12

Yes 10 (48 %) 4 (29 %)

No 8 (38 %) 10 (71 %)

Missing 3(14 %) 0 (0)

**TP53** 0.19

Yes 12 (57 %)  12 (86 %)

No 8 (38 %) 2 (14 %)

Missing 1 (5 %) 0 (0)

**ATRX** 0.77

Yes 17 (81 %) 13 (93 %)

No 3 (14 %) 1 (7 %)

Missing 1 (5 %) 0 (0)
