# Supplementary material for: Plasma alpha B crystallin as potential biomarker for predicting pre-operative seizures in glioma
Source: BMC Neurol. 2024 Jul 6;24:237. doi: 10.1186/s12883-024-03740-x (PMC11227141; doi:10.1186/s12883-024-03740-x)
Supplement: Supplementary file 3 — Supplementary Material 3 [file 12883_2024_3740_MOESM3_ESM.docx]

Supplementary Table 3

Quartile and median data for all the groups

|  | Control | G+E | G-E | IE | E |
| --- | --- | --- | --- | --- | --- |
| 25% Percentile | 149.9 | 112 | 169.4 | 136 | 87.66 |
| Median | 186.5 | 121 | 190.8 | 203.2 | 93.78 |
| 75% Percentile | 218.7 | 143.2 | 250.6 | 237.9 | 114.5 |

Notes: G+E: glioma with epilepsy; G-E: glioma without epilepsy; IE: intractable epilepsy; E Idiopathic epilepsy.
