# Supplementary figures and images for: Plasma alpha B crystallin as potential biomarker for predicting pre-operative seizures in glioma
Source: BMC Neurol. 2024 Jul 6;24:237. doi: 10.1186/s12883-024-03740-x (PMC11227141; doi:10.1186/s12883-024-03740-x)

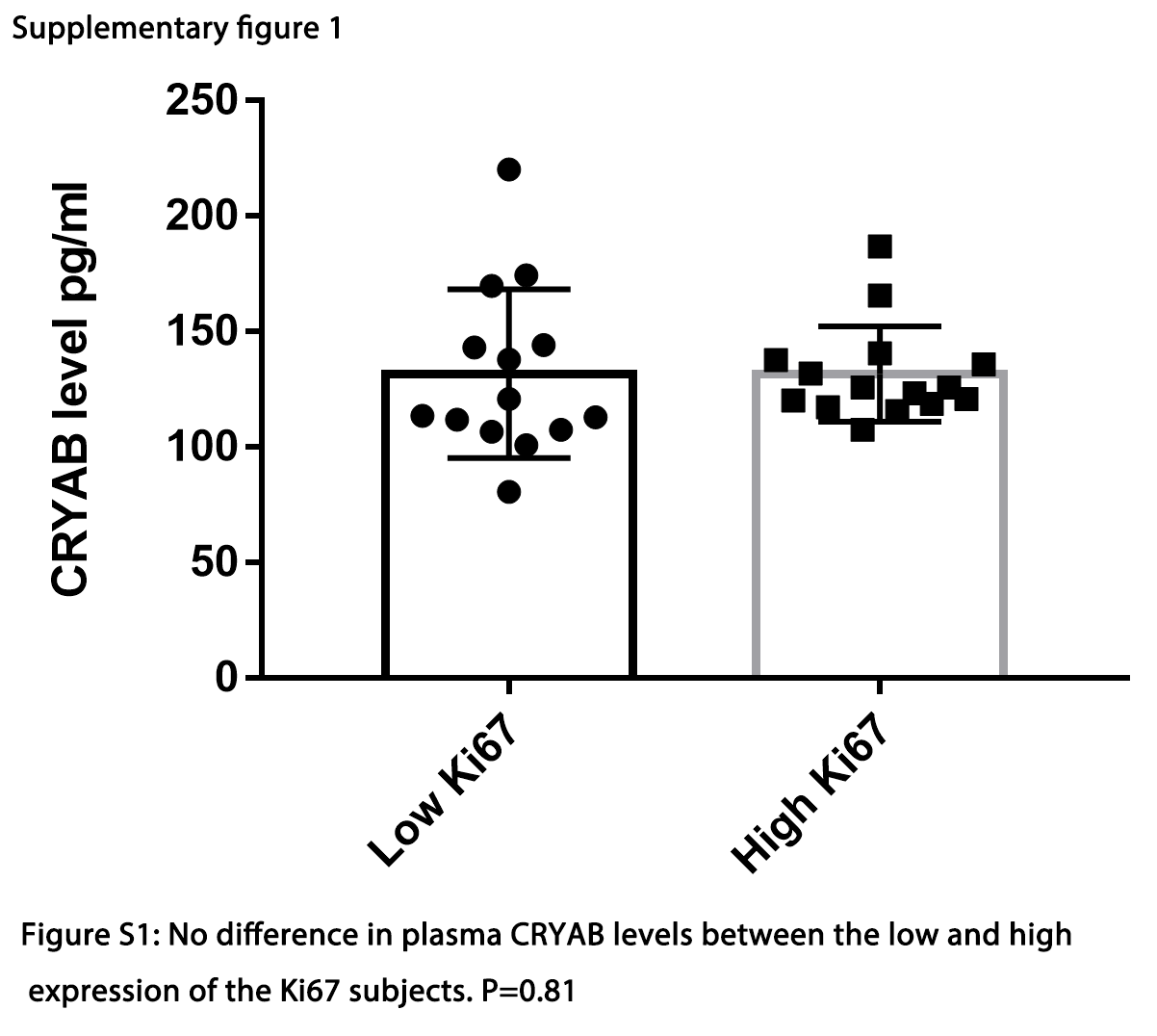

Supplement: Supplementary file 4 — Supplementary Material 4 [file 12883_2024_3740_MOESM4_ESM.tiff]
